# Coinfection of *Salmonella* Typhi and Hepatitis A in a Patient With Prolonged Fever and Jaundice

**DOI:** 10.1155/crdi/6681091

**Published:** 2026-04-16

**Authors:** Mohammad Rasel, Abdullah Al-Jubair, Sudipta Banik, Afsana Mimi

**Affiliations:** ^1^ Department of Medicine, Shaheed Suhrawardy Medical College and Hospital, Dhaka, Bangladesh, suhrawardyhospital.gov.bd; ^2^ Department of Pathology, Army Medical College Bogura, Bogura, Bangladesh

**Keywords:** coinfection, enteric fever, hepatitis A, *Salmonella* Typhi, typhoid

## Abstract

Both *Salmonella* Typhi and hepatitis A infections are endemic in developing countries like Bangladesh. Coinfection of hepatitis A and *S*. Typhi can present with prolonged fever and jaundice. We report a case of coinfection of hepatitis A and *S*. Typhi in a 13‐year‐old child.

## 1. Introduction

Waterborne diseases are a major health concern in developing countries like Bangladesh due to the consumption of contaminated water from local supply authorities, inadequate sewage and drainage infrastructure, and limited access to clean running water, soap, and proper sanitation facilities [1]. Both hepatitis A and typhoid fever are endemic in Bangladesh and are transmitted through the fecal–oral route [2, 3]. A recent nationwide hospital‐based seroprevalence study reported a 19% prevalence of hepatitis A among patients with acute hepatitis [3]. Another study reported that the incidence of *S*. Typhi among blood cultures was 26% [4]. There are few reports of coinfection of these two together, but it is still challenging to determine whether the clinical symptoms are due to acute viral hepatitis or a complication of typhoid hepatitis [5]. Early diagnosis and management of these coinfections become particularly challenging when more than one type of infections coexist with febrile illnesses. Here, we present a case of a 13‐year‐old male child who presented with high‐grade fever for 10 days, along with jaundice. After evaluation, he was diagnosed with a coinfection of hepatitis A and *Salmonella* Typhi.

## 2. Case Presentation

A 13‐year‐old male child got admitted in the inpatient medicine department with a history of high‐grade fever for 10 days and yellowish discoloration of skin for 7 days. The patient was well 10 days before. Then, he developed a high‐grade fever with a highest recorded temperature of 103°F. The fever was not associated with chills or rigor. He also noticed yellowish coloration of his eye and palm for 7 days. There was no history of abdominal pain, bone pain, chest pain, cough, respiratory distress, rashes, gum bleeding, epistaxis, oral ulcer, any features of encephalopathy, nausea, vomiting, and loose stool. He was initially evaluated by a local primary care physician and was treated conservatively with only antipyretics. He had no significant past medical, personal, or travel history.

On general examination, the child was ill looking, febrile (temperature: 102°F), icteric, and mildly dehydrated. The blood pressure was 110/60 mmHg. There was hepatosplenomegaly with a firm, tender liver with smooth, well‐felt margins of 5 cm and a span of 14 cm. A firm spleen was palpable 4 cm below the costal margin. The rest of the physical examinations were within normal limits. Based on the clinical history and physical examination findings, the differential diagnosis considered included acute viral hepatitis, enteric fever with hepatic involvement, leptospirosis, malaria, and viral infections such as *Cytomegalovirus*.

Initial hematologic workup revealed elevated erythrocyte sedimentation rate (ESR) (43 mm in 1^st^ hour). Liver function tests (LFTs) were found markedly deranged with raised hepatic enzymes—serum total bilirubin was 11.20 mg/dL (normal value: < 2 mg/dL), ALT was 730.1 U/L (normal value: < 45 U/L), AST was 394.4 U/L (normal value: < 41 U/L), alkaline phosphatase was 473.1 U/L (normal value: < 150 U/L), and prothrombin time (PT) was 23 s (normal value: 12–17 s). Routine urine examination showed proteinuria (++) and bilirubinuria (+++). All other hematological and biochemical parameters were within normal limits.

Abdominal ultrasound revealed mildly enlarged liver with reduced hepatic parenchymal echogenicity. A mildly enlarged spleen and contracted gallbladder were also seen suggesting acute hepatitis. However, chest X‐ray showed no abnormality in the patient.

Blood was drawn for hepatitis serology and blood culture. Consequently, Anti‐HAV IgM (ELISA) was positive (> 10; cutoff value: < 0.9 negative), but all other hepatitis viruses (B, C, and E) were found negative.

Based on the clinical picture, an initial diagnosis of acute viral hepatitis was made. All supportive treatments (e.g., intravenous fluids, antipyretics, domperidone, and antispasmodic) were prescribed.

However, due to high and prolonged fever, another concomitant infection was suspected. Therefore, intravenous ceftriaxone (100 mg/kg/day) was empirically started on the first day of admission. Subsequently, blood culture report came positive for *Salmonella* Typhi. Antibiogram showed no resistance to any antibiotics.

**FIGURE 1:**
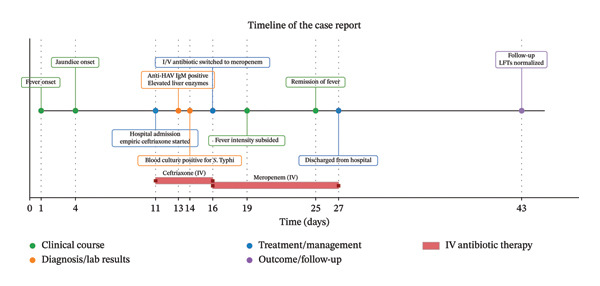
Timeline of the case report.

At this stage, a diagnosis of acute viral hepatitis with enteric fever was confirmed. The child continued to have persistent high‐grade fever despite 5 days of ceftriaxone treatment, which raised concern for possible treatment failure or an additional focus of infection. Hence, intravenous antibiotic was switched to meropenem, which was confirmed sensitive on antibiogram and was available free of cost through the government supply. The addition of oral azithromycin was not considered, as meropenem monotherapy provides similar efficacy [6]. However, in retrospect, given the fully sensitive antibiogram, escalation to meropenem may not have been the most appropriate step. According to antimicrobial stewardship principles, continuation of ceftriaxone or use of another narrow‐spectrum agent with close clinical monitoring could have been considered. With this treatment, the child responded well. His general appearance and appetite started to improve significantly. The patient received parental meropenem for 2 weeks. Remission of fever was appreciated on the 15th day after admission. He had complete resolution of acute viral hepatitis with enteric fever which was evidenced by normal hepatic enzymes during follow‐up at 2 weeks (Figure 1).

## 3. Discussion

*S*. Typhi is a Gram‐negative bacterium which causes systemic disease after transmission through fecal–oral route with contaminated water or food [7]. Following an incubation period ranging from 6 to 30 days, general symptoms of an uncomplicated enteric fever include fever, malaise, anorexia, diarrhea, constipation [8]. On the other hand, hepatitis A is a nonenveloped RNA virus from *Picornaviridae* family which causes self‐limiting infections from consuming contaminated food and water. Prodromal symptoms like fever, malaise, nausea, vomiting, and anorexia can occur about a month following exposure with HAV [9].

Hepatic involvement associated with *S*. Typhi infection is a common manifestation, although severe hepatitis with significant jaundice is less frequent (occurring in about 25% of cases). Hepatitis with elevated liver enzymes is considered as a constant feature of hepatitis A occurring most commonly during the 2nd and 3rd week from the onset of symptoms [10].

Acute viral hepatitis and typhoid hepatitis frequently appear with similar symptoms [11]. Both diseases have an incubation period that ranges from 15 to 60 days, and they may share a common infectious source. Clinically, jaundice should emerge after fever in viral hepatitis, and there is often a 1–7‐day interval between the two. Biochemically, compared to typhoid hepatitis, hepatitis A has significantly higher levels of aminotransferases (greater than 8–10 times the upper limit of normal). The greatest indicator for distinguishing acute viral hepatitis from typhoid hepatitis at admission is an alanine aminotransferase/LDH ratio > 4 in case of acute viral hepatitis [10].

This case report describes a complex clinical scenario in a 13‐year‐old male child with acute viral hepatitis A complicated by enteric fever. This is a coinfection and poses diagnostic and therapeutic challenges that warrant a widespread approach to case management. Acute viral hepatitis is initially presented with high‐grade fever and generalized body ache and jaundice and is consistent with our patient presentation [12]. This diagnosis is further supported by the markedly deranged LFTs, especially by the elevated serum bilirubin and hepatic enzymes. The positive Anti‐HAV IgM indicates current hepatitis A infection, which is the most frequent cause of acute viral hepatitis in children particularly in developing countries [13]. Malaria was not strongly suspected in this case because the child lacked characteristic features such as periodic fever, chills, rigor, anemia, thrombocytopenia, or hemoglobinuria, and there was no travel history in the endemic part of the country. Leptospirosis was considered as a differential diagnosis, but the absence of myalgia, conjunctival suffusion, acute kidney injury, or hypotension made it less likely. However, the persistence of high‐grade fever despite supportive treatment for hepatitis A raised suspicion of a coinfection. This clinical judgment led to the empirical use of antibiotics, which proved crucial in this case. Subsequent positive blood culture for *Salmonella* Typhi confirmed the coinfection with *S*. Typhi and demonstrated the importance of a high index of suspicion of coinfection in atypical or prolonged symptomatic patients [14]. Since the blood culture grew *S*. Typhi and the antibiogram did not show any resistance to antibiotics, escalation to meropenem may not have been required in our case, as fever in patients with typhoid takes a while to subside. From an antimicrobial stewardship perspective, continuation of a sensitive first‐line agent with close clinical monitoring would have been a more appropriate approach. The antibiotic stewardship strategy recommends reassessment at 48–72 h, based on blood culture and susceptibility results [14]. It is also important to note that the local antibiogram data should inform the choice of empiric therapy and help reserve the use of carbapenems for confirmed cases of multidrug‐resistant infections [14].

This case report emphasizes that clinicians should not overlook more than one infection in patients with complicated clinical pictures in areas where both diseases are highly prevalent. This case also highlights the public health challenges faced by low‐income countries, including inadequate water, sanitation, and hygiene (WASH). Lack of safe and sufficient WASH contributes to high incidence of fecal–orally transmitted diseases like hepatitis A and *S*. Typhi. Poor WASH conditions account for more than one million deaths every year. Under the Sustainable Development Goals (SDGs), Member States of the United Nations (UN) aim to improve global water and sanitation by 2030, emphasizing the need for universal access to WASH [15]. Measures such as vaccination against typhoid and hepatitis A are also particularly important in regions with high disease prevalence [16, 17].

## 4. Conclusion

Hepatitis A and *S*. Typhi coinfection should be considered in patients with prolonged fever with jaundice, especially in endemic areas. Coinfection should be suspected when fever does not subside with the appearance of jaundice and evidence of hepatitis. Accurate identification of the etiology is essential to avoid treatment failure in coinfection cases.

## Author Contributions

Mohammad Rasel: writing–original draft, review, and editing. Abdullah Al‐Jubair: writing–original draft, review, and editing. Sudipta Banik: writing–original draft. Afsana Mimi: writing–original draft.

## Funding

The authors received no financial support for authorship and/or publication of this case report.

## Ethics Statement

Ethics approval is not required for de‐identified single case reports based on institutional policies.

## Consent

Written informed consent was obtained from the patient to publish this case report in accordance with the journal’s patient consent policy. A copy of the written consent is available for review by the editor‐in‐chief of this journal on request.

## Conflicts of Interest

The authors declare no conflicts of interest.

## Data Availability

Data sharing is not applicable to this article as no datasets were generated or analyzed during the current study.
